# Sinularin Induces Apoptosis through Mitochondria Dysfunction and Inactivation of the pI3K/Akt/mTOR Pathway in Gastric Carcinoma Cells

**DOI:** 10.3390/md14080142

**Published:** 2016-07-27

**Authors:** Yu-Jen Wu, Bing-Sang Wong, Shu-Hao Yea, Chi-I Lu, Shun-Hsiang Weng

**Affiliations:** 1 Department of Food Science and Nutrition, Meiho University, Pingtung 91202, Taiwan; x00002180@meiho.edu.tw; 2 Department of Beauty Science, Meiho University, Pingtung 91202, Taiwan; 3 Antai Medical Care Cooperation Antai Tian-Sheng Memorial Hospital, Pingtung 92842, Taiwan; a094162@mail.tsmh.org.tw; 4 Yu Jun Biotechnology Co., Ltd., Kaoshiun 91202, Taiwan; a714825936@gmail.com; 5 Department of Nursing, Meiho University, Pingtung 91202, Taiwan; x00002177@meiho.edu.tw

**Keywords:** sinularin, gastric cancer, apoptosis, mitochondrial dysfunction

## Abstract

Sinularin is an active compound isolated from the cultured soft coral *Sinularia flexibilis*. In this study, we investigated the effects of sinularin on two human gastric cancer cell lines, AGS and NCI-N87. Our results demonstrated that sinularin suppressed the proliferation of gastric cancer cells in a dose-dependent manner and induced apoptosis. In addition, the loss of mitochondrial membrane potential, the release of cytochrome *C*, the activation of Bax, Bad and caspase-3/9, and the suppression of *p*-Bad, Bcl-xL and Bcl-2 were observed in the cells treated with sinularin. This finding suggests that sinularin-induced apoptosis is associated with mitochondria-mediated apoptosis and occurs through caspase-dependent pathways. Furthermore, sinularin inhibited the phosphoinositol 3-kinase/Akt/mechanistic target of the rapamycin signaling pathway. Taken together, our results show that sinularin-induced apoptosis is mediated by activation of the caspase cascade and mitochondrial dysfunction. Our findings suggest that sinularin merits further evaluation as a chemotherapeutic agent for human gastric cancer.

## 1. Introduction

According to a recent report from the World Health Organization (WHO), gastric cancer is the fifth most common cancer worldwide and was the third leading cause of cancer death [1]. Evidence from epidemiological studies has shown that *Helicobacter pylori* infection is the main cause of gastric cancer [1]. Because its symptoms are often nonspecific, diagnosis is often delayed until metastasis occurs. In addition, the prognosis of the disease is generally poor because it mostly affects elderly patients (average age: 70–75 years). Therefore, the five-year survival rate is less than 10% [2].

Phosphoinositol 3-kinase (PI3K)/Akt/mechanistic target of rapamycin (mTOR) signaling is a crucial pathway involved in cell survival and differentiation, proliferation, apoptosis and metastasis [3,4]. Active Akt binds to the related G protein-coupled receptors or receptor tyrosine kinase of growth factors such as epidermal growth factor and insulin-like growth factor, generating phosphatidylinositol triphosphate (PIP3) at the plasma membrane. PIP3 binds to the pleckstrin homology domain of Akt, resulting in the translocation of Akt to the membrane. Activation of PI3K occurs when PI3K is recruited to the phosphotyrosine residues of its ligand through its Src Homology 2 domains. Activated PI3K then phosphorylates the inositol ring of phosphatidylinositol bisphosphate (PIP2) to generate PIP3. The signaling phospholipid PIP3 thereby regulates cell survival, growth, and morphological change [5,6]. The cellular phosphatase and tensin homolog (PTEN) hydrolyzes PIP3 to generate PIP2, which regulates cell proliferation by reducing PIP3 production [7,8]. Overexpression of PI3K and inactivation of the *PTEN* gene activate the PI3K signaling pathway, which may cause cancer [5,9,10].

Several recent studies have demonstrated that 11-dehydrosinulariolide, 13-acetoxysarcocrassolide and 11-*epi*-sinulariolide acetate isolated from soft corals promote apoptotic induction against cell migration and exhibit anti-tumor effects [10,11,12]. Moreover, a quinone derivative, flexibilisquinone, derived from the cultured soft coral *Sinularia flexibilis*, exhibits anti-inflammatory effects [13]. In addition, sinularin extracted from the soft coral *Sinularia flexibilis* possesses significant inhibitory effects against cell proliferation and migration in A2058 melanoma cells [14].

It is vital to explore new effective anticancer drugs and develop therapies for gastric cancer. In this study, we examined the cytotoxic effect of sinularin isolated from the soft coral *S. flexibilis* on the gastric carcinoma cell lines AGS and NCI-N87. Sinularin possessed antiproliferative and apoptosis-inducing activities against AGS and NCI-N87 cells. These results showed that sinularin inhibits cell proliferation and induces apoptosis through mitochondria-dependent apoptosis and inhibition of the PI3K/Akt/mTOR pathway. These results provide useful information for understanding the biochemical aspects of the cytotoxic effects of sinularin on AGS and NCI-N87 cells and will accelerate drug development and progression of monitoring of human gastric carcinoma.

## 2. Materials and Methods

### 2.1. Cell Lines

The AGS cell line (ATCC: CRL-1739) is a poorly-differentiated human gastric carcinoma cell line [15] and the NCI-N87 cell line (ATCC: CRL-5822) is the liver metastasis of well-differentiated human gastric cancer cell line [16]. Both cell lines were purchased from the Food Industry Research Development Institute in Taiwan (Hsinchu, Taiwan). AGS cells were maintained in the Ham’s F12 medium (Corning, New York, NY, USA) and NCI-N87 cells were maintained in the RPMI-1640 medium (Corning). Both of the media were supplemented with 10% fetal calf serum (FCS) (Gibco, Waltham, MA, USA), 4.5 g/L glucose, 4 mM l-glutamine, 1.5 g/L sodium bicarbonate, 1 mM sodium pyruvate, 100 μg/mL streptomycin and 100 units/mL penicillin, and placed in a 5% CO_2_ incubator at 37 °C.

### 2.2. Reagents

**Figure 1:**
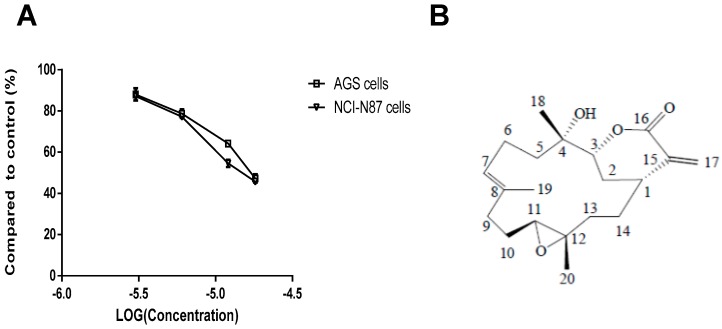
Effect of sinularin on the cell viability of AGS and NCI-N87 human gastric cancer cell lines. (**A**) The cells were incubated with sinularin at 3, 6, 12, 18 μM for 24 h, and the cytotoxic effect of sinularin on the cells was analyzed using a 3-(4,5-cimethylthiazol-2-yl)-2,5-diphenyl tetrazolium bromide (MTT) assay; (**B**) Chemical structure of sinularin.

In this study, sinularin was obtained from National Museum of Marine Biology & Aquarium (Pintung, Taiwan) and the chemical structure showed in Figure 1B. The anti-β-actin antibody was obtained from Sigma-Aldrich (St. Louis, MO, USA), apoptosis antibody sample kit, pro-apoptosis bcl-2 family antibody sample kit, pro-survival Bcl-2 family antibody sample kit, phospho-pI3 kinase p85 (Tyr458)/P55 (Tyr799) antibody, phosopho-GSK3β (Ser9) (D3A4) rabbit antibody, PI3 kinase p110α antibody were obtained from Cell Signaling (Danvers, MA, USA), anti-cytochrome *C*, CD95/Fas, AIF, phospho-Akt1, Akt1, mTOR/FRAP and phosopho-mTOR (Phospho-Ser2481) antibody were obtained from Epitomics (Bellerica, MA, USA). Goat anti-rabbit and horseradish peroxidase conjugated IgG was obtained for Millipore (Bellerica, MA, USA).

### 2.3. MTT Assay

Cells were seeded on a 24-well plate at 1 × 10^5^ cells/well. After adherence, the cells were treated with different concentrations of sinularin for 24 h. After the cell culture medium was removed, the cells were washed three times with phosphate-buffered saline (PBS), and 200 μL of 1 mg/mL 3-(4,5-cimethylthiazol-2-yl)-2,5-diphenyl tetrazolium bromide (MTT) obtained from Sigma-Aldrich (St. Louis, MO, USA) were added to each well and incubated at 37 °C for 15 min. The solution in each well was then removed, and 200 μL of Dimethyl sulfoxide (DMSO) were added to each well to dissolve the formazan crystals. The absorption intensity was analyzed using an ELISA plate reader at 590 nm, and the results were used to calculate the viability of the cells.

### 2.4. Cell Migration Assay

Cells at 1 × 10^5^ cells/well in 300 μL of the culture medium without FCS were seeded in a 24-well Transwell culture insert (BD Biosciences, San Jose, CA, USA), and 700 μL of the culture medium containing FCS was added to the bottom of each well. After 24-h incubation in a 5% CO_2_ incubator at 37 °C, the cells were washed two or three times with PBS and then fixed in 100% methanol for 10 min, followed by Giemsa staining for 1 h. After washing with distilled water, the specimens were dried and mounted onto a slide for cell counting under a microscope.

### 2.5. Cell Apoptosis Assay

#### 2.5.1. Annexin V/Propidium Iodide (PI) Stain

The Annexin V-FITC Apoptosis Detection Kit (Strong Biotech Corporation, Taipei, Taiwan) was used for Annexin V/propidium iodide (PI) staining according to the manufacturer’s instructions. Briefly, cells were seeded in a six-well plate at 3 × 10^5^ cells/well. After adhesion, the cells were treated with different concentrations of sinularin for 12 h and harvested by trypsinization. Cells were centrifuged at 2000 rpm for 5 min, and the cell pellet was resuspended in 1 mL of PBS and transferred to a 1.5 mL Eppendorf tube. The cells were washed three times with PBS and then mixed with 100 μL Annexin V binding buffer, followed by the addition of 5 μL each of Annexin V and PI. After incubation for 10–15 min in the dark, Annexin V binding buffer was added to bring the total volume to 1 mL. The cells were then transferred into a flow cytometry tube and analyzed using a flow cytometer (Cytomics FC 500, Beckman Coulter, Atlanta, GA, USA)

#### 2.5.2. TUNEL/DAPI Stain

TUNEL/DAPI staining was performed using the In Situ Cell Death Detection Kit, Fluorescein (Roche Diagnostics, Indianapolis, IN, USA), and DAPI (Sigma-Aldrich, St. Louis, MO, USA). The gastric cancer cell lines were cultured on coverslips at 1 × 10^4^ cells/well. After adhesion, the cells were treated with different concentrations of sinularin for 24 h. After removal of cell culture media, the cells were fixed with 4% paraformaldehyde for 20 min at room temperature. The cells were then washed three times with PBS, and the cell membranes were permeabilized with 0.1% Triton-X 100 for 2 min on ice. Subsequently, the cells were washed three times with cold PBS. Thirty microliters of precooled ice-cold TUNEL staining solution and enzyme mixed at a 1:19 ratio were added to the cells, followed by incubation for 1 h at 37 °C. The cells were washed three times with PBS, and the cells were stained with 1 μL of DAPI in 1 mL of PBS for 10 s. The samples were mounted onto slides and examined under a fluorescent microscope.

#### 2.5.3. JC-1 Stain

The JC-1 stain assay was performed using a MitoProbe JC-1 Assay Kit (ThermoFisher, Waltham, MA, USA). Briefly, gastric cancer cell lines were cultured on coverslips at 1 × 10^4^ cells/well. After adherence, the cells were treated with different concentrations of sinularin for 24 h. The cell culture media were removed and the cells were incubated with 100 μL JC-1 stain solution (200 μM) for 15–20 min in a 5% CO_2_ incubator at 37 °C. The samples were then washed 3 times with PBS and the cells on coverslips were mounted onto slides and examined under a fluorescent microscope.

## 3. Results

### 3.1. Sinularin Inhibited Gastric Cancer Cell Growth and Cell Migration

In this study, an MTT assay was used to evaluate the cytotoxic effect of sinularin on AGS and NCI-N87 gastric cancer cell lines. Sinularin at the concentrations of 3, 6, 12, and 18 μM exerted a dose-dependent effect on these two cell lines. Cell viability decreased with the increasing sinularin concentration (Figure 1). The effectively reduced cell proliferation of sinularin on AGS cells is 52% (the IC_50_: 17.73 μM) and on NCI-N87 cells it is 53.5% (the IC_50_: 15.13 μM).

**Figure 2:**
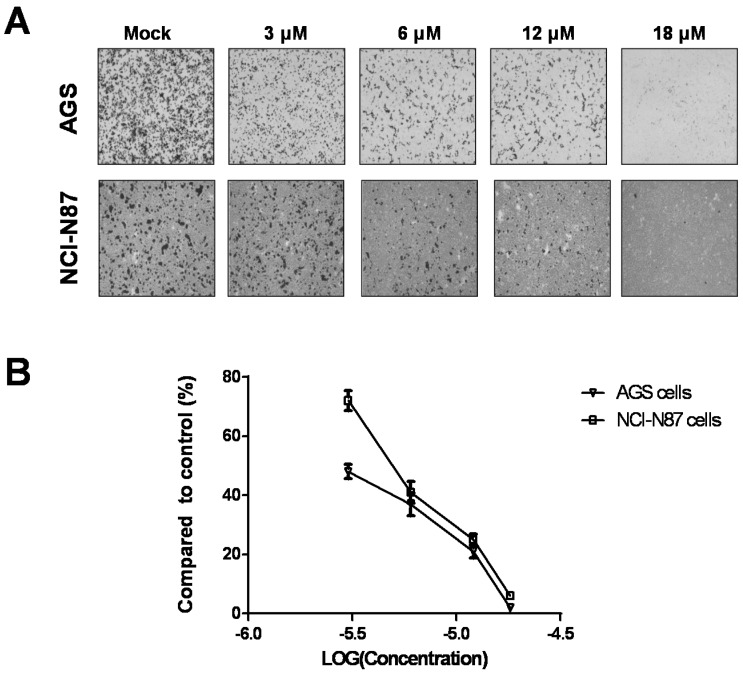
Effect of sinularin on gastric cancer cell migration. (**A**) Cells were treated with different concentrations of sinularin for 24 h, and the cell migration in these two cell lines was analyzed (magnification: 600×); (**B**) Data from triplicate experiments.

This study used a Transwell cell migration assay to evaluate whether sinularin potentially inhibits gastric cancer cell migration. The results demonstrated that sinularin dose-dependently inhibited AGS and NCI-N87 gastric cancer cell migration (Figure 2). Interestingly, our results showed that low doses of sinularin treatment had no significant cytotoxic effect on the cells, while the inhibition of cell migration was observed at low concentrations. Notably, the inhibition of cell migration by sinularin was more significant in AGS cells compared to NCI-N87 cells (Figure 2).

### 3.2. Sinularin Induced Apoptosis in Gastric Cancer Cell Lines

**Figure 3:**
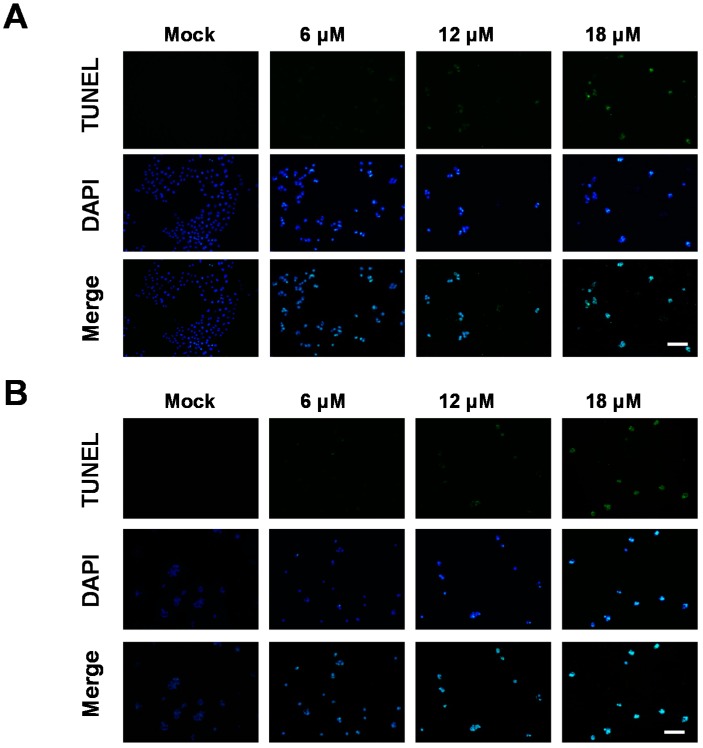
Analysis of sinularin-induced apoptosis by TUNEL/DAPI staining. (**A**) AGS and (**B**) NCI-N87 human gastric cancer cell lines. The cells were incubated with sinularin at 6, 12 and 18 μM for 24 h, and then stained with TUNEL/DAPI staining. Cell shrinkage and apoptotic bodies were observed in the cells with sinularin treatment. Scale bars = 50 nm.

**Figure 4:**
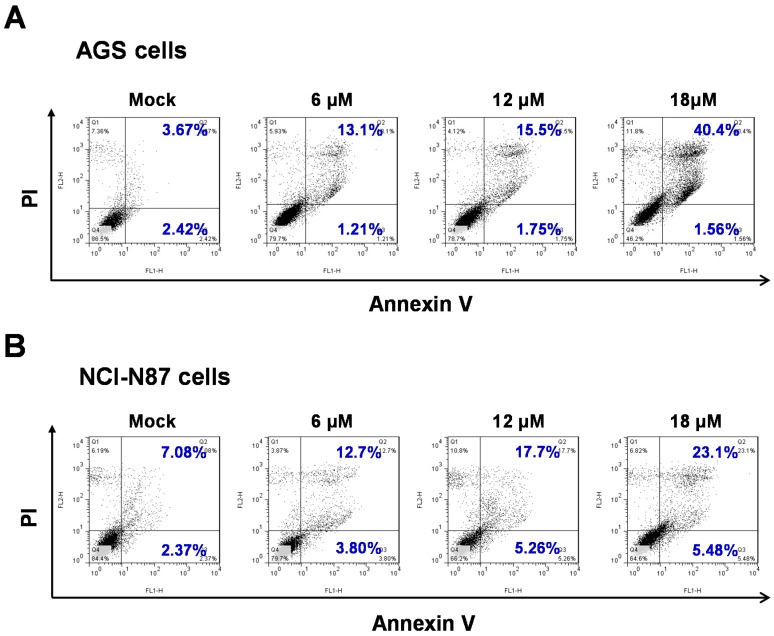
Sinularin-induced apoptosis in AGS and NCI-N87 human gastric cancer cell lines. (**A**) AGS cells; (**B**) NCI-N87 cells. Both of the cells were incubated with sinularin at 6, 12 and 18 μM for 24 h, and then stained with Annexin V and PI. Fluorescence intensities were detected by flow cytometry in order to determine the effects of sinularin on earlier apoptosis and late apoptosis.

To examine whether the cytotoxic effect of sinularin is mediated by apoptosis, TUNEL staining was used to evaluate apoptosis in the cells after sinularin treatment. In addition, DAPI staining revealed the shrinkage of cells treated with 6 μM sinularin, and apoptotic bodies were present in the samples treated with 12 μM sinularin (Figure 3). To further investigate sinularin-induced apoptosis in detail, a flow cytometric method that detects apoptotic cells by FITC-Annexin V/PI was employed. In the AGS cells, the early apoptosis:untreated cell ratio was 2.42%, and that in cells treated with 18 μM sinularin was 1.56%. Moreover, the late apoptosis:untreated cell ratio was 3.6%, and that in cells treated with 18 μM sinularin dramatically increased to 40.4%. In the NCI-N87 cells, the early apoptosis:untreated cell ratio was 2.37%, and that in cells treated with 18 μM sinularin was 5.48%. In addition, the late apoptosis:untreated cell ratio was 7.08%, and that in cells treated with 18 μM sinularin increased to 23.1% (Figure 4).

### 3.3. Sinularin-Induced Apoptosis Was Associated with Decreased Mitochondrial Membrane Potential in Human Gastric Cancer Cells

**Figure 5:**
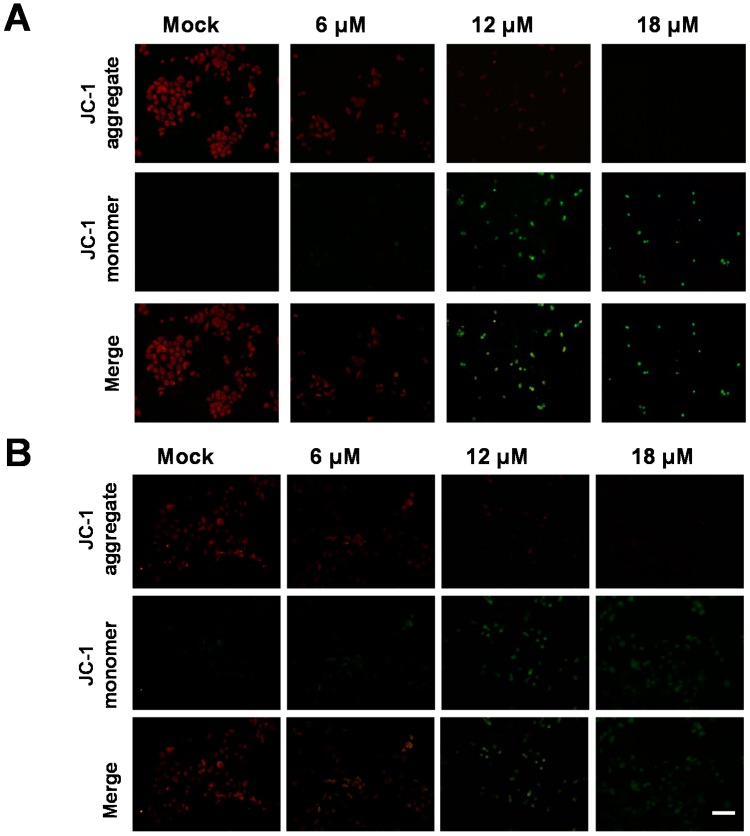
JC-1 staining to examine the changes in the mitochondrial membrane potential in (**A**) AGS and (**B**) NCI-N87 gastric cancer cells after sinularin treatment. Cells were treated with sinularin (6, 12, 18 μM) for 24 h, and then incubated with JC-1 staining solution. The results showed that the red fluorescence intensity decreased and the green fluorescence intensity increased in both cell lines, indicating that sinularin caused changes in the mitochondrial membrane potential that induced mitochondria dysfunction. Scale bar = 50 nm.

External and internal stress may result in decreases in the mitochondrial membrane potential in cells, which also induces apoptosis. In this study, changes in the mitochondrial membrane potential were detected using JC-1 staining. As positively charged molecules, JC-1 enters the negatively charged mitochondria in healthy cells, and aggregated JC-1 exhibits red fluorescence (580 nm). However, when the mitochondrial membrane potential changes, as observed in early apoptotic cells, JC-1 is converted into monomers and exhibits green fluorescence (530 nm). As shown in Figure 5, with increasing sinularin concentration, the red fluorescence intensity decreased and the green fluorescence intensity increased in cells. These results demonstrated that sinularin reduced the mitochondrial membrane potential in AGS and NCI-N87 cells, thereby potentially leading to mitochondrial dysfunction.

**Figure 6:**
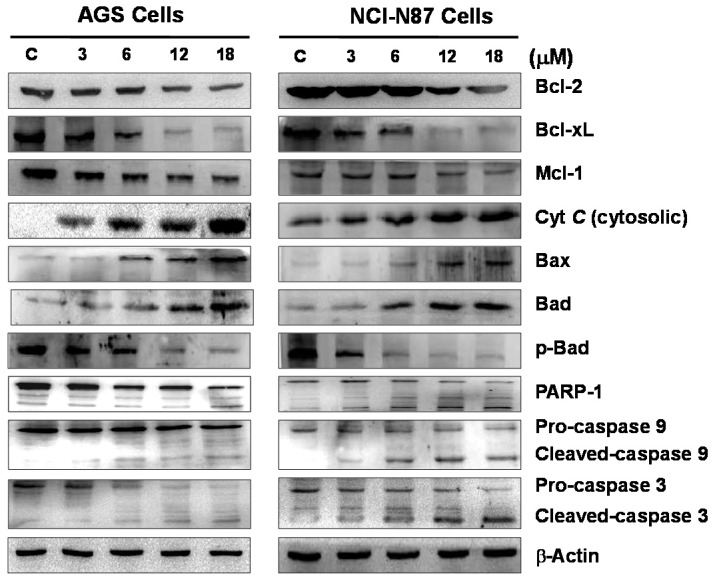
Western blotting of mitochondria-related apoptotic proteins in gastric cancer cells with sinularin treatment. AGS and NCI-N87 human gastric cancer cells were treated with sinularin (3, 6, 12 and 18 μM), and the protein expression of the prosurvival Bcl-2 family (Bcl-2, Bcl-xL, Mcl-1, and Bad) and the proapoptosis Bcl-2 family (Bax and Bad) was analyzed. Western blotting showed that the expression of the proteins of the prosurvival Bcl-2 family decreased, and the expression of the proteins of the proapoptosis Bcl-2 family increased. The protein expression of cytochrome *C* increased. The protein expression of the downstream procaspase-9 decreased, whereas the protein expression of cleaved caspase-9 and cleaved PARP-1 increased. β-actin was used as a loading control.

This study demonstrated that sinularin affects the mitochondrial membrane potential. To understand the underlying mechanisms, in this study, Western blotting was used to analyze apoptotic proteins involved in mitochondrial dysfunction in AGS and NCI-N87 cells. The analysis revealed that with the increasing sinularin concentration, the expression of the prosurvival Bcl-2 family (Bcl-2, Bcl-xL, Mcl-1, and *p*-Bad) decreased, and the expression of the proapoptotic Bcl-2 family (Bax and Bad) increased. When mitochondrial damage occurs, cytochrome *C* and AIF are released into the cytoplasm, and cytochrome *C* may activate downstream caspases, causing apoptosis. Our results showed that the protein level of cytochrome *C* in the cells increased with the increasing sinularin concentration and treatment period. By contrast, the protein expression of the downstream procaspase-9 and procaspase-3 decreased, and the protein expression of the cleaved caspase-9 and cleaved caspase-3 increased. In addition, the protein expression of cleaved poly [ADP-ribose] polymerase 1 (PARP-1) increased. Because cleavage of PARP-1 induces DNA breaks, our results indicate that sinularin-induced apoptosis is mediated by mitochondrial dysfunction (Figure 6).

### 3.4. Effect of Sinularin on the PI3K/Akt/mTOR Pathway in Gastric Cancer Cells

**Figure 7:**
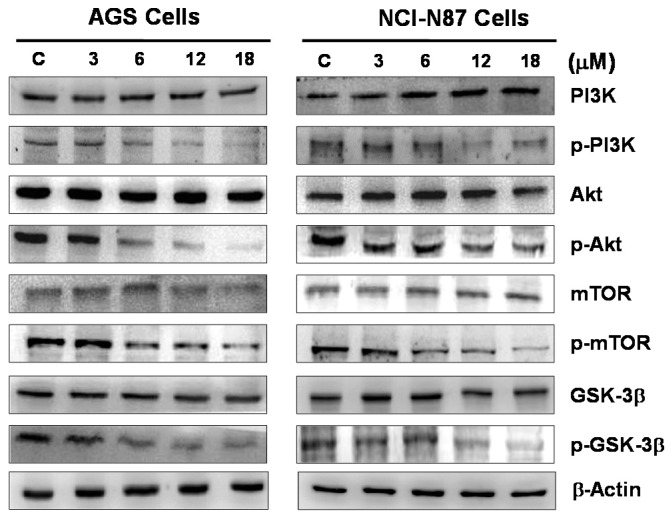
Western blotting of key proteins in the PI3K/Akt/mTOR pathway in cells with sinularin treatment. AGS and NCI-N87 human gastric cancer cells were treated with sinularin (3, 6, 12 and 18 μM), and the protein expression of PI3K, Akt, mTOR, and GSK3β was analyzed. The expression of phosphorylated PI3K, Akt, mTOR, and GSK3β decreased with increasing sinularin concentrations. β-actin was used as a loading control.

The PI3K/Akt/mTOR pathway plays a key role in the regulation of many physiological and biochemical functions in cells, including cell growth, proliferation, and division. GSK3β activity mediates Akt activation, which may regulate the cell cycle and apoptosis. Therefore, this study investigated whether the effect of sinularin on tumor cell proliferation is associated with the PI3K/Akt/mTOR pathway. By using Western blotting, our study found that the expression levels of phosphorylated PI3K, Akt, mTOR, and GSK3β decreased with the increasing sinularin concentration. By contrast, the total protein expression of PI3K, Akt, and mTOR remain unchanged (Figure 7).

## 4. Discussion

This study demonstrated that sinularin exerts an anticancer effect on gastric cancer cells, which is mainly mediated by its cytotoxic effect and apoptosis induction. Two gastric cancer cell lines, AGS and NCI-N87 cells, were examined for the effects of sinularin treatments. Although the AGS cells are known as being the poorly differentiated cells while the NCI-N87 cells are well-differentiated cells, as expected, both of the cells demonstrated a reduction of cell migration and an increase of cell apoptosis in response to the sinularin treatments. Our results showed a similar effect of sinularin treatment on the reduction of cell proliferation of both AGS (52%) and NCI-87 (53.5%). Similarly, the inhibitory effect of cell migration by sinularin was shown on both cell lines. In many of the anti-tumor treatments we studied, these natural compounds exhibited similar effects on several cancer cell lines, indicating that these compounds indeed affected both cell proliferation and metastasis so as to trigger the anti-tumor mechanism. Several studies have reported that apoptosis may be induced through an intrinsic or extrinsic pathway [17,18,19], and activation of the caspase cascade is also involved in apoptosis [20]. Activation of caspase occurs only when caspase is cleaved, and activation of initiator caspases, such as caspase-8 and caspase-9, is required for activation of executioner caspase (i.e., caspase-3) for inducing apoptosis. In our study, sinularin treatment led to the activation of caspase-3 and caspase-9. Whether an intrinsic or extrinsic pathway is the key pathway inducing apoptosis in our model is still unknown and requires further investigation. However, the executioner caspase, caspase-3, was activated, which led to the induction of apoptosis. The results suggest that sinularin-induced apoptosis involves an intrinsic pathway that is associated with mitochondrial dysfunction.

Mitochondria-dependent apoptosis is regulated by the proteins of the Bcl-2 family. The expression of proteins of the prosurvival Bcl-2 family, such as Bcl-2, Bcl-xL, Mcl-1, and *p*-Bad, is inhibited when cells are under certain stresses; consequently, proapoptotic proteins, such as Bad and Bax, are activated. The changes in the expression of the Bcl-2 family result in calcium ion flow imbalance, leading to mitochondrial dysfunction and the release of cytochrome *C* and AIF into the cytoplasm. Consequently, cytochrome *C* binds to caspase-9 and apoptosis protease-activating factor-1, further activating caspase-3 for apoptosis induction [21,22]. In addition, activated caspase-3 cleaves PARP to generate activated PARP. Activated PARP has been shown to cause chromosome condensation and DNA fragmentation [23]. In addition, cleaved PARP-1-induced apoptosis has been linked to Bax and calpains, leading to AIF-mediated programmed cell death [24,25]. Our results showed that sinularin promotes the expression of proapoptotic proteins (Bax and Bad) and inhibits the expression of antiapoptotic proteins (Bcl-2, Bcl-xl, Mcl-1 and *p*-Bad) (Figure 6). Furthermore, cells with sinularin treatment exhibited a lower mitochondrial membrane potential, which led to cytochrome *C* release. Moreover, the expression of activated caspase-3 and caspase-9 and cleaved PARP increased with the increasing sinularin concentration (Figure 6). The results suggest that sinularin induces apoptosis in human gastric cancer cells through mitochondrial dysfunction.

Studies have shown that Akt is a serine/threonine kinase that can phosphorylate more than 9000 proteins [26,27]. Therefore, Akt is a key factor in the regulation of cell growth. Activated Akt may be present in the cytoplasm or may enter the nucleus to regulate antiapoptotic proteins and cell proliferation [28]. Akt can also increase the phosphorylation of Bax, which leads to inactivation of Bad, thus inhibiting apoptosis [29]. mTOR can increase protein translation through the activation of Akt, thereby promoting cell growth. The AKT-regulated mTOR pathway is complex and has a multistep mechanism. Tuberous sclerosis 1 (TSC1) and TSC2 exist as a complex in the cells, and the TSC1-TSC2 complex can inactivate Rheb, leading to mTOR inactivation. By contrast, activation of Akt can inhibit the activity of TSC2, which promotes Rheb accumulation and results in mTOR activation [30,31]. In addition, mTOR can phosphorylate 4E-BP1 to free 4E-BP1 from the mRNA cap-binding protein eIF-4E, which allows 4E-BP1 to bind to the translation initiation complex and initiate protein synthesis [32]. GSK3β, a multifunctional kinase, is the primary target of Akt. It regulates various cellular processes, including cell proliferation, apoptosis, cell cycle, glycogen metabolism and protein synthesis [33,34]. GSK3β also plays a role in the regulation of key molecules of the Wnt pathway. Activated Akt phosphorylates GSK3β, which decreases the activity of GSK3β, resulting in cell growth. By contrast, when Akt is inactivated, GSK3β can cause a β-catenin breakdown through the phosphorylation of β-catenin, which inhibits the Wnt pathway [35,36].

Several studies have reported that the PI3K/Akt/mTOR signaling pathway is involved in cell proliferation, differentiation, survival and metastasis [37]. PI3K activation can also activate Akt [38]. Akt, a serine/threonine kinase, phosphorylates Bad when activated. Phosphorylated Bad can inhibit apoptosis. Therefore, this observation suggests that PI3K is involved in the regulation of apoptosis. In addition, mTOR may increase protein translation through Akt activation, and protein synthesis and cell growth are induced by the phosphorylation of mTOR [39,40]. Furthermore, as the primary target protein of Akt, GSK3β plays a key role in the regulation of apoptosis and the cell cycle [34].

## 5. Conclusions

**Figure 8:**
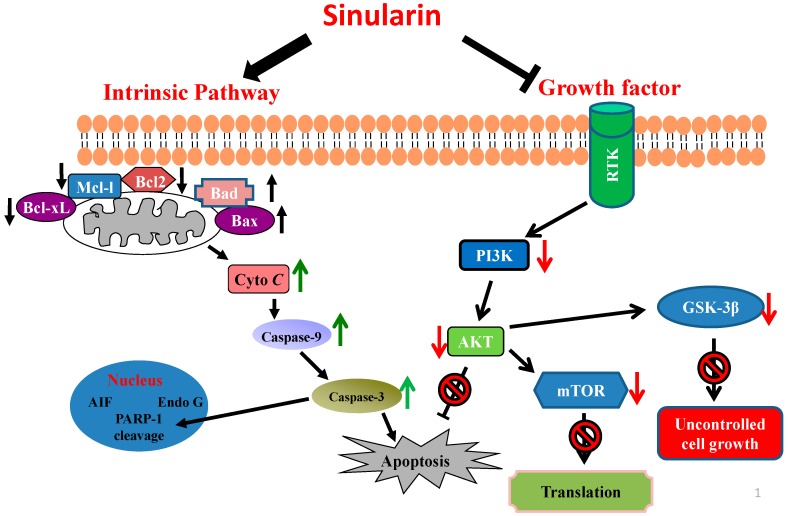
Sinularin-induced apoptosis in the AGS and NCI-N87 gastric cancer cells. The anticancer effect of sinularin is mediated by the induction of mitochondria dysfunction and the inhibition of the PI3K/Akt/mTOR signaling pathway.

The results of our study demonstrated that the expression of phosphorylated PI3K, Akt, mTOR, and GSK3β proteins decreased with the increasing sinularin concentration. Our findings suggest that sinularin inhibits gastric cancer cell proliferation and induces apoptosis through mitochondrial dysfunction and inhibition of the PI3K/Akt/mTOR signaling pathway (Figure 8).
